# Radioprotective Effects and Mechanisms of One-Year and Seven-Year White Tea Extracts Against ^137^Cs Radiation-Induced Cell Damage

**DOI:** 10.3390/molecules30071448

**Published:** 2025-03-25

**Authors:** Chen Xia, Meisheng Cai, Yanting Lu, Bingkui Wang, Linglin Xu, Kaixi Wang, Zhonghua Liu

**Affiliations:** 1 Department of Tea Science, College of Agriculture and Biotechnology, Zhejiang University, Hangzhou 310058, China; 2 Fuding Tea Industry Development Leading Group, Ningde 355200, China; 3 Institute of Crops and Nuclear Technology Utilization, Zhejiang Academy of Agricultural Sciences, Hangzhou 310021, China

**Keywords:** one-year white tea, seven-year white tea, ionizing radiation, cesium, anti-apoptosis, metabolomics

## Abstract

Ionizing radiation (IR) is widely present in the environment, with ^137^Cesium (Cs) radiation having particularly severe impacts during nuclear accidents. The objective of our study was to assess the radiation protection or repair effect of one year (WT-1Y) or seven years (WT-7Y) of storage on white teas, as well as to investigate the mechanism of radioprotection. HGC-27 cells exposed to ^137^Cs γ-rays (30 Gy) exhibited significant changes in cell structure, apoptosis, ROS, LDH, and their expression of p53 and Caspase-3. The results showed that WT-1Y and WT-7Y acted as antioxidants, showed reduced ROS and LDH levels, and had increased CAT and SOD activities as well as cell survival rate. The WT treatments significantly inhibited apoptosis in both the pre- and post-radiation groups, with WT-1 showing stronger effects in pretreatment by reducing LDH, p53, and Caspase-3 levels and enhancing ROS scavenging and enzyme activities. Post-treatment analysis revealed WT-7 had greater effects on cell viability and SOD activity. Overall, both WT-1 and WT-7 mitigated radiation damage, likely by inhibiting the p53/Caspase-3 apoptosis pathway. A Spearman analysis of the differential metabolites in WT-1Y and WT-7Y with cellular radioprotective indicators revealed that metabolites, such as EGC, procyanidin B4, and phenolic acids (abundant in WT-1Y), quercetin-3-glucosylrutinoside, and caffeine (enriched in WT-7Y) contributed to their distinct effects in the pre- and post-treatment of ^137^Cs γ-rays.

## 1. Introduction

With increasing exposure to environmental, medical, and industrial ionizing radiation (IR), the demand for effective natural radioprotectors is growing. Cs isotopes, particularly the radioactive ^137^Cs, are among the most widely dispersed contaminants following nuclear accidents [1,2]. These isotopes permeate the atmosphere, soil, and water and accumulate in biological systems through the food chain, posing significant risks to human health [3,4]. Additionally, the growing use of medical diagnostic technologies, such as CT scans and X-rays, has further elevated public radiation exposure. Ionizing radiation (IR) is a series of energy waves that cause direct or indirect damage to key biomolecules like DNA, proteins, and lipids [5]. It has been found that the indirect effects of IR in cells are more likely to occur, as a cell contains nearly 70% water [6]. At the molecular level, the cytotoxic effects of IR on cells are largely attributed to primary species produced by the radiolysis of water, such as hydroxyl radicals (•OH) and hydrogen peroxide (H_2_O_2_) [7]. These reactive oxygen species (ROS) result in the damage or death of cells, which causes DNA chain breakage, protein carboxylation, and lipid peroxidation [8]. Overexposure to ionizing radiation might damage healthy tissues and organs, leading to weakened immunity, skin diseases, and even cancer [9]. In recent years, with the increasing use of radiation, the nuclear safety situation has become increasingly tense. Radiation protection has once again attracted people’s attention. Compounds with anti-inflammatory, antioxidant, antibacterial, immunomodulatory, free radical scavenging, and anti-stress properties might act as potential radioprotectors [10]. In previous studies, natural products such as polyphenols, flavonoids, saponins, and polysaccharides have shown good effects of protection against radiation [11,12,13,14,15].

White tea is one of the traditional tea types in China and originates from the silky white feathers covering unripe leaves and buds. It contains various compounds, including alkaloids, polyphenols, flavonoids, amino acids, proteins, polysaccharides, vitamins, organic acids, etc. [16]. Recent studies have revealed that the components/compounds of white tea have antioxidant, anti-obesity, antidiabetic, and anticarcinogenic and antimutagenic activities [17,18,19,20].

In our previous research, we found that white tea extract had a significant effect on the oxidative stress injury of lung fibrosis induced by nano-sized SiO_2_ in rats. A white tea extract treatment group and an epigallocatechin gallate (EGCG) showed significant alleviation in the content of nitrogen monoxide (NO), inflammatory factor Interleukin-6 (IL-6), Glutathione peroxidase (GSH-Px) activity in the lungs of the rats [21]. White tea extracts could obviously improve cigarette smoke-induced chronic obstructive pulmonary disease (COPD) in mice by antioxidation, anti-inflammation, and the regulation of NO abnormalities. Both white tea extracts and EGCG treatment could significantly reduce malondialdehyde (MDA), IL-6, and tumor necrosis factor-alpha (TNF-*α*) levels and up-regulate SOD activity [22].

White tea has been proven to have a maximum level of polyphenols that is even higher than that in green tea, especially catechins and their derivatives [23]. Therefore, it has a superior free radical scavenging ability. White tea extract has shown a strong scavenging ability for 2,2-Diphenyl-1-picrylhydrazyl (DPPH) and 2,2′-Azino-bis(3-ethylbenzothiazoline-6-sulfonic acid) (ABTS) free radicals, and has protected against H_2_O_2_-induced DNA damage [20]. The long-term intake of white tea could protect the kidney from oxidative stress and prevent oxidative tissue damage [24,25].

It is commonly believed that storage could improve the flavor and commercial value of white tea. Meanwhile, the chemical composition of white tea changes with longer storage times. Dai et al. found that a novel type of substance named 8-C N- ethyl-2-pyrrolidinone-substituted flavan-3-ol (EPSF) forms during white tea storage, and that its contents are highly related to storage time [26]. With the increase in years of storage, the content of tea polyphenols decreases, while the content of flavonoids increases [27]. The changes in chemical composition in the aging process might lead to changes in their bioactivity as well. However, until now, no reports have been found concerning the radioprotective activity of white tea changes after a long time in storage.

According to the characteristics of white tea, it may have the potential to act as a natural radioprotector. This study aims to investigate the effect of different storage times of white tea on its bioactivity in human gastric cells (HGC-27) after pre- and post-treatment with ^137^Cs radiation. Therefore, we studied the difference between the metabolites of white tea stored for 1 and 7 years, respectively, and investigated their roles and mechanisms of radioprotective activity, including the reparation of radiation-induced cellular damages. This study provides a scientific basis for understanding the influence of white tea storage time on its radioprotective activity.

## 2. Results

### 2.1. Metabolite Differences Between WT-1Y and WT-7Y

**Figure 1:**
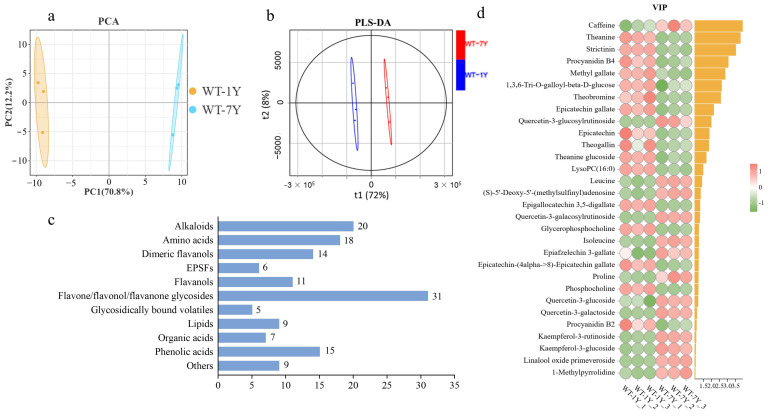
Metabolite differences between WT-1Y and WT-7Y. (**a**). Principal Component Analysis (PCA) score plot showing the separation of metabolites between WT-1Y (orange) and WT-7Y (blue); (**b**). Partial Least Squares Discriminant Analysis (PLS-DA) plot indicating distinct clustering of WT-1Y (red) and WT-7Y (blue) based on their metabolomic profiles. (**c**). Bar chart depicting the number of metabolites classified into 11 different categories. (**d**). Heatmap of differential metabolites identified by Variable Importance in Projection (VIP) scores greater than 1 and statistically significant (*p* < 0.05, *t*-test).

To obtain a detailed understanding of the metabolite changes in white tea during storage, the metabolic profiles of WT-1Y and WT-7Y were analyzed. After peak extraction and alignment, a total of 145 metabolites were detected. A PCA of these metabolites (Figure 1a) demonstrated distinct clustering and separation trends between WT-1Y and WT-7Y, and the principal components 1 and 2 explained 70.8% and 12.2% of the total variances, respectively. As shown in Figure 1b, the PLS-DA plot also indicated that after 7 years of aging, the composition and abundance of the metabolites in white tea significantly changed.

A total of 145 metabolites were classified into 11 classes (Figure 1c), which included 20 alkaloids, 18 amino acids, 14 dimeric flavanols, 6 EPSFs, 11 flavanols, 31 flavone/flavonol/flavanone glycosides, 5 glycosidically bound volatiles, 9 lipids, 7 orange acids, 15 phenolic acids, and 9 others. The VIP score was used to identify the substances that contributed to the distinctions in the PLS-DA model. A higher VIP value represented a more significant contribution of separation. Thirty differential metabolites (VIP > 1, *p* < 0.05 by *t*-test) were identified in WT-1Y and WT-7Y (Figure 1d). The data revealed significant changes in the substance levels after 7 years of storage. Specifically, 14 differential metabolites, including caffeine, quercetin-3-glucosylrutinoside, and leucine, increased by 1.17-, 3.24-, and 1.21-fold, respectively, compared to their levels in WT-1Y. Conversely, 16 differential metabolites, such as theanine, strictinin, and procyanidin B4, decreased to 79.09%, 78.16%, and 62.42%, respectively, of their original contents in WT-1Y.

**Figure 2:**
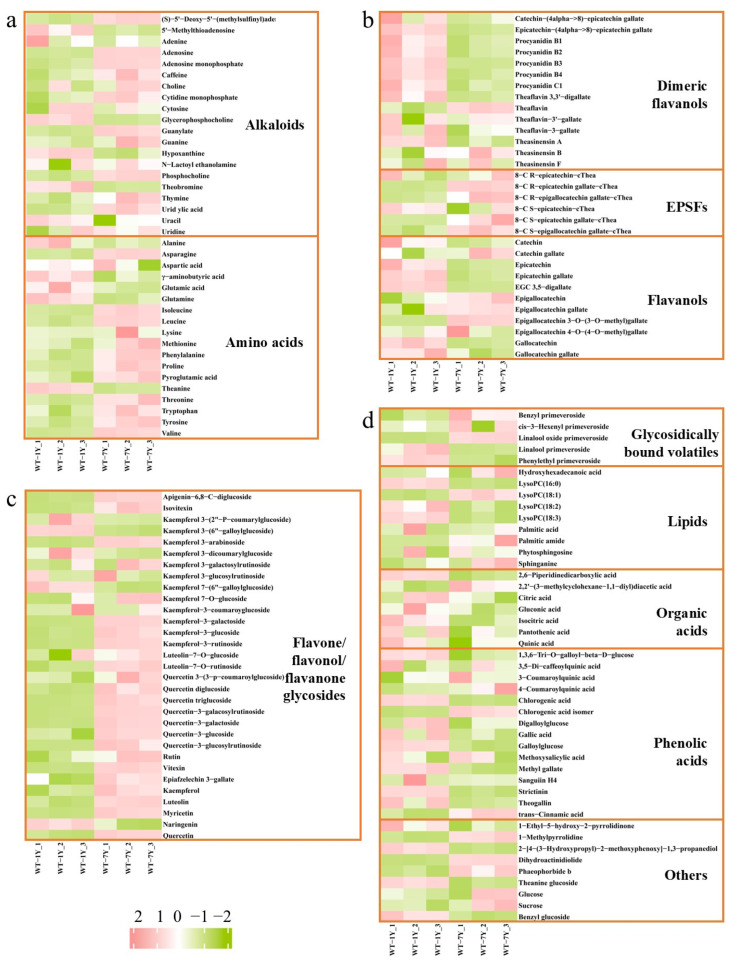
Metabolite abundance of WT-1Y and WT-7Y. (**a**). Heatmap showing the relative abundance of alkaloids and amino acids in WT-1Y and WT-7Y. (**b**). Heatmap illustrating the abundance of dimeric flavanols, EPSFs (8-C N-ethyl-2-pyrrolidinone-substituted flavan-3-ols), and flavanols in both WT-1Y and WT-7Y. (**c**). Heatmap representing the relative abundance of flavone, flavonol, and flavanone glycosides. (**d**). Heatmap showing the metabolite abundance in categories including glycosidically bound volatiles, lipids, organic acids, phenolic acids, and others.

A heatmap analysis revealed changes in the metabolite abundance of WT after 7 years of aging (Figure 2). In the heatmap, red represents high and green represents low degrees of change. After 7 years in storage, most flavone/flavonol/flavanone glycosides and EPSFs increased, while dimeric catechins, flavanols, organic acids, and phenolic acids decreased.

### 2.2. WT-1Y and WT-7Y Extracts Reduce Radiation-Induced Cytotoxicity

**Figure 3:**
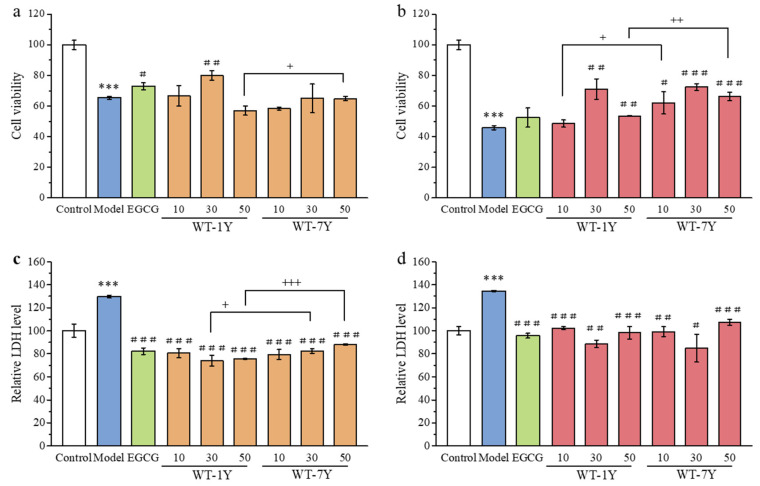
Post-radiation viability and LDH contents of HGC-27 cells pre- (**a**,**c**), and post- (**b**,**d**) treatments with different dosages (10, 30, 50 mg/L) of WT-1Y and WT-7Y extracts by exposure to IR (30 Gy) (n = 3). Student’s *t*-test was used for significance analysis. *** *p* < 0.001 compared with control; ^#^ *p* < 0.05, ^##^ *p* < 0.01, ^###^ *p* < 0.001 compared with model; ^+^ *p* < 0.05, ^++^ *p* < 0.01, ^+++^ *p* < 0.001 compared between WT-1Y and WT-7Y at same concentration. The white column represented the Control group, the blue column represented the Model group, the green column represented the EGCG group, the orange column represented the pre-treatment of WT, and the red column represented the post-treatment of WT.

CCK-8 staining and LDH content detection were used to evaluate radiation-induced cytotoxicity on HGC-27 cells. The effect of WT-1Y and WT-7Y extracts on the cell viability was ascertained by absorption reads after CCK-8 staining, which was positively correlated with the number of live cells. LDH, as an enzyme present in the cytoplasm, is released into the extracellular space when cells are damaged or dead [28]. Therefore, LDH is often used as a monitoring indicator for cell damage [29]. As shown in Figure 3, HGC-27 cells irradiated at a dose of 30 Gy showed a significant decrease in cell viability and an increase in released LDH content. Both pre- and post-treatment, cell viability first increased and then decreased with the increase in WT extract concentration. The administration of 30 mg/L WT-1Y and WT-7Y showed the best cell protection effect compared to the other concentrations used because the cells had the highest cell viability and least LDH released (Figure 3a,b). The pre-treatment with 30 mg/L of WT-1Y and WT-7Y extracts increased the viability of the irradiated cells by 16.63% and 1.75%, respectively, compared to the model group. Similarly, the post-treatment with 30 mg/L of WT-1Y or WT-7Y extracts increased the irradiated cell viability by 25.21% and 26.50%, respectively.

Figure 3c,d showed that irradiation significantly increased LDH release compared to the model group. The pre-treatment with 30 mg/L of WT-1Y and WT-7Y extracts reduced LDH release by 43.29% and 36.99%, respectively, compared to the model group. The post-treatment resulted in a greater reduction, with WT-1Y and WT-7Y reducing LDH release by 44.11% and 46.66%, respectively, demonstrating their protective effects against radiation-induced cytotoxicity, which were better than EGCG. Comparing the protective effects of the WT-1Y and WT-7Y extracts at the same concentration, we found that WT-1Y showed better protection pre-treatment, while WY-7Y showed better protection post-treatment.

### 2.3. WT-1Y and WT-7Y Extracts Rebalance the Radiation-Induced Changes in Intracellular ROS, SOD, and CAT Levels

**Figure 4:**
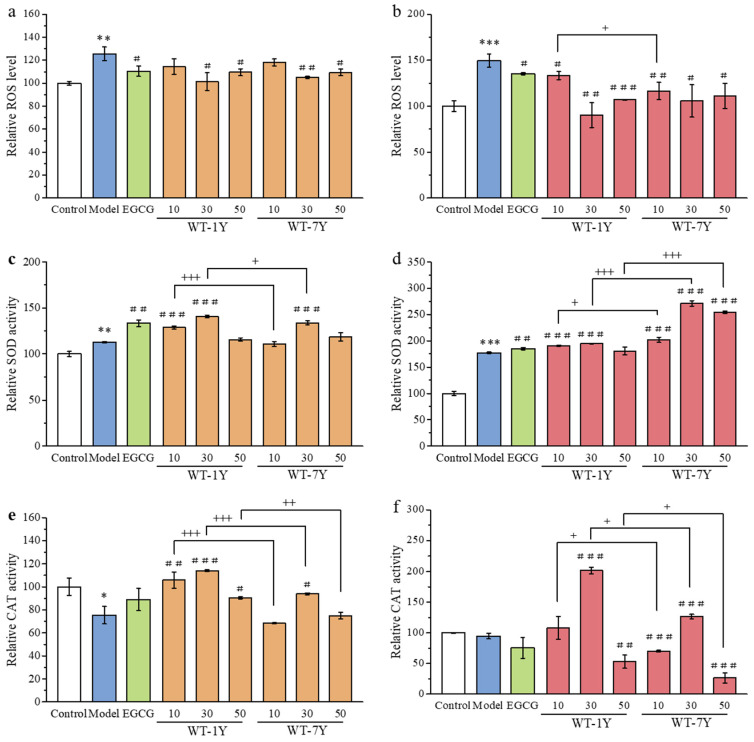
The intracellular ROS level and SOD and CAT activity of HGC-27 cells pre- (**a**,**c**,**e**) and post- (**b**,**d**,**f**) treatments with the different concentrations (10–50 mg/L) of WT-1Y and WT-7Y under exposure to IR (30 Gy) (n = 3). A Student’s *t*-test was used to analyze statistical significance. * *p* < 0.05, ** *p* < 0.01, *** *p* < 0.001 compared with the control; ^#^ *p* < 0.05, ^##^ *p* < 0.01, ^###^ *p* < 0.001 compared with the model; ^+^ *p* < 0.05, ^++^ *p* < 0.01, ^+++^ *p* < 0.001 compared between WT-1Y and WT-7Y at the same concentration. The white column represented the Control group, the blue column represented the Model group, the green column represented the EGCG group, the orange column represented the pre-treatment of WT, and the red column represented the post-treatment of WT.

IR is known to induce a notable disturbance in oxidative status. To further investigate the effects of WT-1Y and WT-7Y extracts on irradiation-damaged cells, the levels of intracellular ROS were measured. The ROS levels in HGC-27 cells were measured by DCFH-DA, which emits fluorescence upon oxidation by ROS, and the intensity of this fluorescence is directly proportional to the level of ROS. As shown in Figure 4a,b, the ROS levels in the HGC-27 cells significantly increased when the cells were exposed to 30 Gy irradiation. Pre- or post-treatment with WT-1Y and WT-7Y could alleviate the oxidative stress status in irradiated cells. During both pre- and post-treatment, the ROS scavenging ability first increased and then decreased with increasing concentrations of WT extracts. The 30 mg/L dosage was most effective in reducing ROS production. Pre-treatment with 30 mg/L of WT-1Y and WT-7Y resulted in a ROS scavenging effect, reducing ROS levels by 24.20% and 20.67%, respectively. Post-treatment with 30 mg/L of WT-1Y and WT-7Y demonstrated stronger ROS scavenging activity, reducing ROS production by 59.31% and 43.90%, respectively. Comparing the ROS scavenging ability of WT-1Y and WT-7Y extracts at the same concentration, we found no significant difference in the pre-treatment, while WT-7Y showed superior performance in the post-treatment.

The rebalancing of the ROS level was mainly related to the activity of antioxidant enzymes. As shown in Figure 4c–f, the SOD and CAT activities of HGC-27 cells with different treatments were measured. During pre-treatment, the administration of WT extracts promoted the activity of SOD. A dosage of 30 mg/L had the best promoting effect in the WT-1Y and WT-7Y groups. Compared to the WT-7Y group, WT-1Y group had higher SOD activity at the same concentration. The variation pattern of CAT activity in the pre-treatment was the same as that of SOD. The CAT activity of the WT-1Y group was 1.55, 1.22, and 1.21 times higher than that of the WT-7Y group at concentrations of 10, 30, and 50mg/L, respectively. In the post-treatment, the administration of WT extracts also promoted the activity of SOD and CAT enzymes. At a dosage of 30 mg/L, the administration of WT-1Y extracts promoted the activities of SOD and CAT by 1.94- and 2.02-fold, respectively, while the administration of WT-7Y extracts elevated the activities of SOD and CAT 2.71- and 1.26-fold, respectively. Although all model groups received the same radiation treatment, significant differences in SOD and CAT activity were observed between the groups, which may be attributed to individual biological variations, cell types, or the cells’ response to radiation. After radiation exposure, the increase in SOD activity may be due to the cells’ enhanced superoxide dismutase activity to cope with the accumulation of superoxides, while the decrease in CAT activity may be related to the accumulation of hydrogen peroxide. They respond to radiation damage through different mechanisms and have varying sensitivities to radiation, which may lead to differences in activity. Under treatment with EGCG, WT-1Y, and WT-7Y, the 30 mg/L dose increased CAT activity, which may effectively alleviate oxidative stress. However, the 10 mg/L dose was too low, and the 50 mg/L dose was too high, neither of which effectively improved the antioxidant capacity, leading to a decrease in CAT activity. This indicates that the effect of dosage on cellular antioxidant capacity is dose dependent.

### 2.4. WT-1Y and WT-7Y Extracts Maintain the Integrity of Cellular Ultrastructure

**Figure 5:**
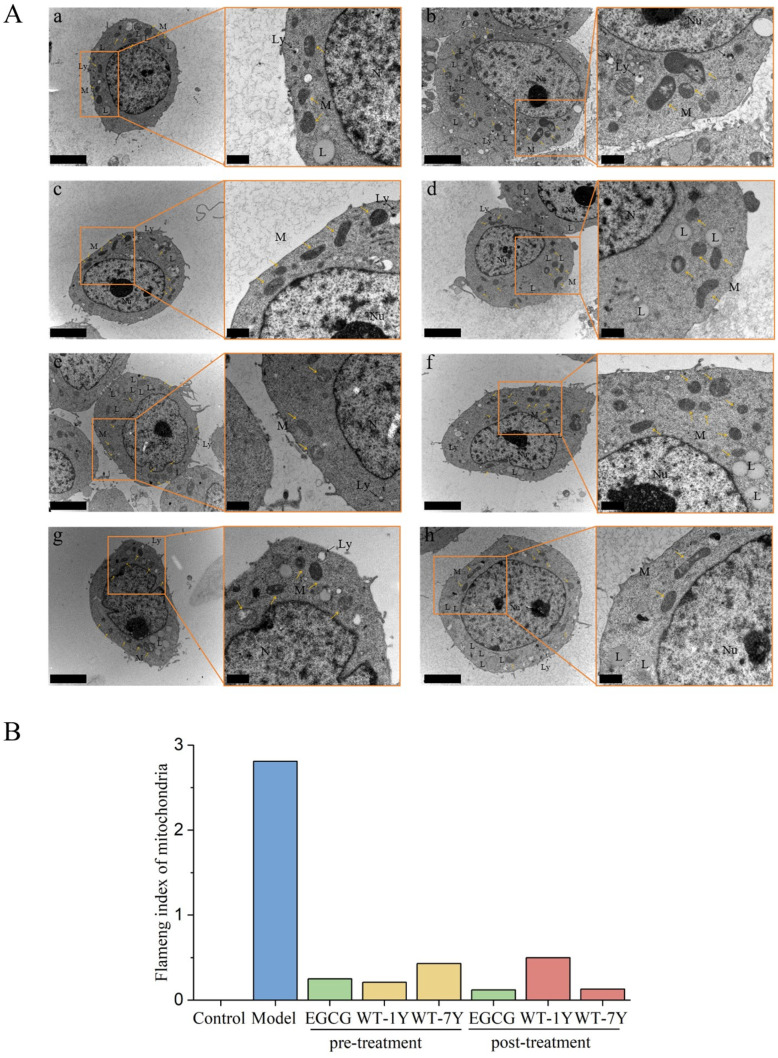
Ultrastructure of HGC-27cells (**A**) and Flameng index of mitochondria (**B**). In (**A**), Control untreated HGC-27 cells (**a**); model exposed to 30 Gy IR (**b**); pre-treatments with EGCG (**c**), 30 mg/L of WT-1Y (**d**) and 30 mg/L of WT-7Y (**e**); post-treatments with EGCG (**f**), 30 mg/L of WT-1Y (**g**) and 30 mg/L of WT-7Y (**h**). Nucleus (N), nucleolus (Nu), lipid (L), mitochondria (M, yellow arrow), lysosome (Ly, black arrow). In (**a**–**h**), the left images had a 4-μm scale bar, and the right images were magnified views with a 1-μm scale bar. In (**B**), The Flameng index of the Control group was 0. The blue column represented the Model group, the green column represented the EGCG group, the orange column represented the pre-treatment of WT, and the red column represented the post-treatment of WT.

Radiation treatment caused significant damage to HGC-27 cells, particularly in terms of their organelles. Transmission electron microscopy (TEM) analysis revealed marked morphological changes in the mitochondria of the model group cells, including the rupture of mitochondrial cristae and vacuolization, indicating damage to membrane integrity (Figure 5). An accumulation of lipid droplets and an increase in lysosomes were also observed, reflecting metabolic dysregulation and autophagic response as the cells cope with radiation-induced oxidative stress. These changes typically indicate lipid peroxidation, with the resultant lipid peroxidation products accumulating within the cell as lipid droplets, while the increase in lysosomes suggests that the cells are enhancing autophagic processes to clear damaged cellular components in response to radiation damage.

The pre- and post-treatments with white tea extracts (WT-1Y and WT-7Y) significantly alleviated these damages, as evidenced by a marked reduction in lipid droplet and lysosome count, as well as an improvement in mitochondrial morphology. At the dose of 30 mg/L, the WT extracts effectively mitigated oxidative stress through their antioxidant properties, reducing lipid peroxidation and alleviating lipid droplet accumulation. Furthermore, the white tea extracts reduced ROS generation and modulated autophagic activity, leading to a decrease in lysosome numbers and lessening the need for excessive cellular clearance. The protective effect of white tea extracts on mitochondria was also demonstrated by a reduction in vacuolization and rupture, thereby maintaining mitochondrial structural integrity.

The extent of mitochondrial damage was further quantified using the Flameng index [30]. The Flameng index of the Control group was 0, which indicated that the mitochondrial structure was completely normal. In the radiation-treated model group, the Flameng index significantly increased, reaching a high value close to 3, indicating severe mitochondrial damage. This damage was characterized by the swelling of some mitochondria, with a significant reduction in matrix density and separation of the cristae. In more severe cases, mitochondrial cristae were broken and the matrix exhibited coagulation. After treatment, the mitochondrial structure was generally restored to normal, with only occasional mild swelling observed. This result further confirms the significant role of WT extracts in preserving mitochondrial integrity. Both the pre-treatment and post-treatment with WT-1Y and WT-7Y, as well as EGCG, showed similar effects in protecting cellular organelles, suggesting that all three have potential as radiation protectants, helping cells cope with radiation-induced oxidative damage and maintaining cellular function.

### 2.5. WT-1Y and WT-7Y Inhibit Radiation-Induced Activation of p53 and Caspase-3

**Figure 6:**
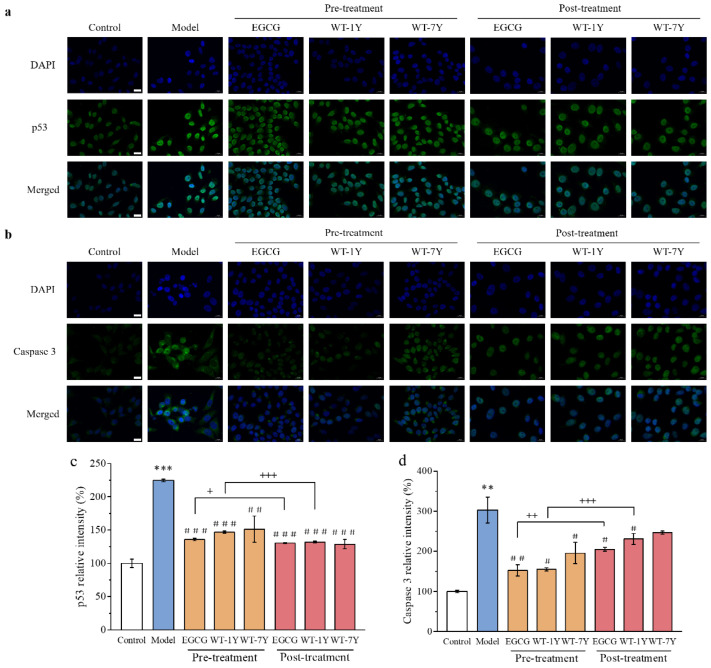
Fluorescence intensity image and relative level for p53 (**a**,**c**) and caspase 3 (**b**,**d**) in HGC-27 cells after pre-and post-treatments with 30 mg/L of WT-1Y and WT-7Y by exposure to IR (30 Gy) (Scale bar = 20 µm, n = 3). Student’s *t*-test was used for significance analysis. ** *p* < 0.01, *** *p* < 0.001 compared with control; ^#^ *p* < 0.05, ^##^ *p* < 0.01, ^###^ *p* < 0.001 compared with model; ^+^ *p* < 0.05, ^++^ *p* < 0.01, ^+++^ *p* < 0.001 compared between EGCG, WT-1Y, and WT-7Y with different treatments. The white column represented the Control group, the blue column represented the Model group, the orange column represented the pre-treatment, and the red column represented the post-treatment.

From the perspective of enhancing cell protection and rebalancing ROS levels, 30 mg/L of WT extract demonstrated the most optimal effect; therefore, this concentration was chosen for further mechanistic studies. p53 and caspase 3 are closely related to cell apoptosis. Figure 6 shows that p53 and caspase 3 were activated in the irradiated HGC-27 cells, as the fluorescence intensity was 2.25 and 3.03 times higher, respectively, compared to the control group. WT-1Y in the pre- and post-treatments inhibited the activation of p53, resulting in a reduction of 78.02% and 73.45% in p53 activity, respectively. Similarly, pre- and post-treatment with WT-7Y inhibited the activation of p53. However, there was no significant difference in the inhibitory effect between WT-1Y and WT-7Y. Meanwhile, adding WT-1Y in the pre- and post-treatments could inhibit the activation of caspase 3. Comparing the two processing methods, we found that pre-treatment had a more significant inhibitory effect on caspase 3 expression, while post-treatment had a more significant inhibitory effect on p53 expression.

### 2.6. Analysis of the Correlation Between the Level of Intracellular Indexes and Metabolite Contents of WT-1Y and WT-7Y

**Figure 7:**
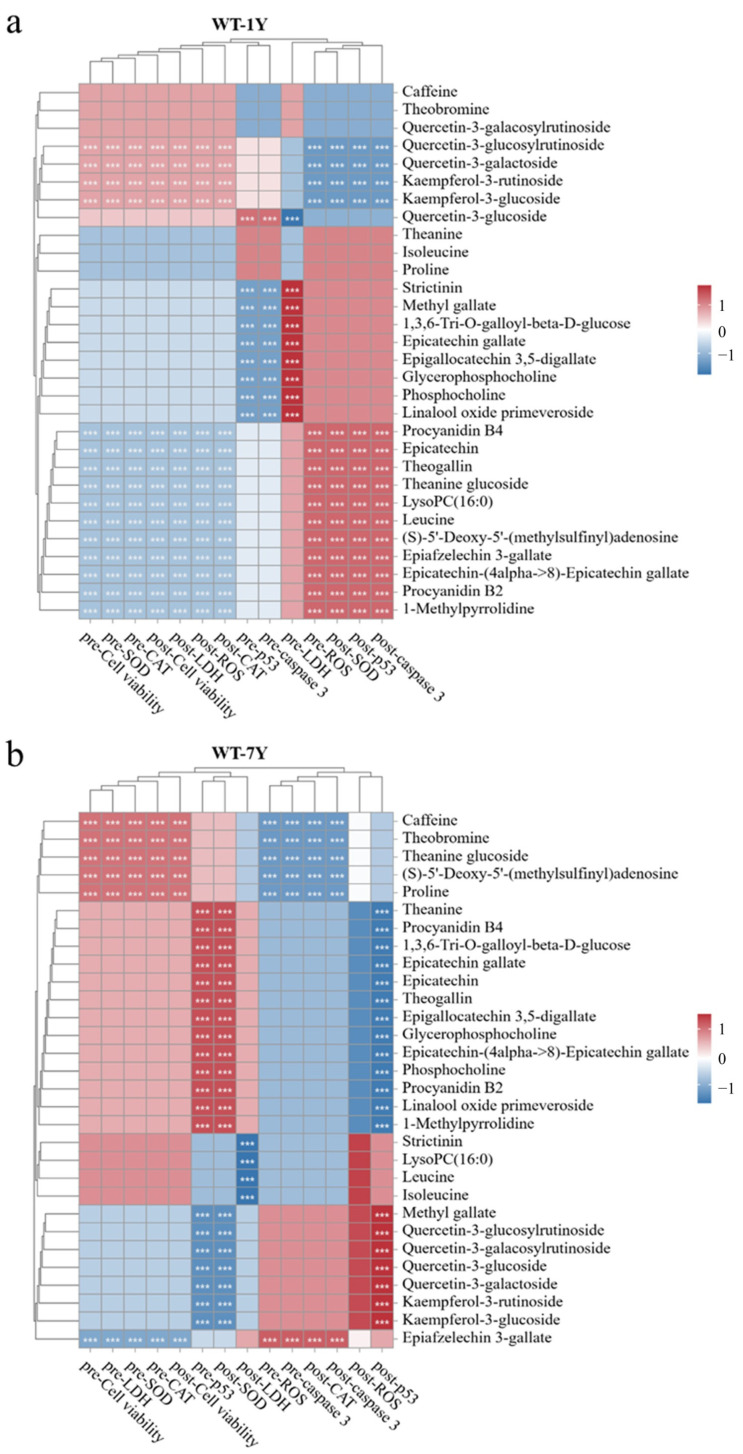
Correlation between the level of cell viability, LDH release, ROS production, SOD and CAT activity, and p53 and caspase-3 expression and differential metabolite contents of WT-1Y (**a**) and WT-7Y (**b**) in pre- and post-treatments with a dosage of 30 mg/L. Each row represents a different parameter or metabolite, and each column represents different treatment conditions (pre-treatment or post-treatment). Asterisks indicate significance (***  p  <  0.001) using Spearman’s correlation analysis.

When the HGC-27 cells were exposed to 30 Gy of radiation, the administration of 30 mg/L WT-1Y and WT-7Y extracts showed a significant impact on cell viability, LDH release, ROS production, SOD and CAT activity, and p53 and caspase-3 expression in pre- and post-treatments. Therefore, indicators at this dosage were selected for further correlation analysis with metabolomics. Figure 7 shows that there was a positive or negative correlation between the differential metabolites and indexes. Different metabolites played varying roles in the WT-1Y and WT-7Y groups. Due to variations in abundance, the same substance may exhibit distinct functions in either the WT-1Y or WT-7Y group. For example, in the WT-1Y group, many metabolites, including strictinin, methyl gallate, 1,3,6-Tri-O-galloyl-beta-D-glucose, and epicatechin gallate (EGC), had a negative correlation with p53 and caspase 3 contents in pre-treatment. Caffeine was correlated with cell viability, the LDH release, ROS production, SOD and CAT activity, and caspase-3 expression in the WT-7Y group, whereas there was no correlation in the WT-1Y group.

## 3. Discussion

Ionizing radiation pollution poses serious threats to human and ecosystem safety, with nuclear contamination introducing radioactive ^137^Cs into the ecological chain, thereby increasing exposure risks [1,2]. Developing safe and effective natural radioprotectors is critical for mitigating radiation-induced damage and safeguarding public health. With potent antioxidant, anti-inflammatory, and immunomodulatory properties, white tea extract functions both as a healthful beverage and an effective tool to mitigate environmental radiation exposure [31]. This study evaluated the protective effects of WT-1Y and WT-7Y on cells under pre- and post-treatments with ^137^Cs radiation, focusing on their roles in antioxidant activity, mitochondrial protection, and anti-apoptotic effects. Metabolomic analysis revealed key differential metabolites associated with radiation protection, shedding light on the multilevel protective mechanisms of white tea extracts.

Radiation exposure rapidly elevates intracellular ROS levels, triggering oxidative stress that causes lipid peroxidation, mitochondrial dysfunction, and DNA breaks, ultimately leading to cellular dysfunction and apoptosis [7]. Exposure to 30 Gy ^137^Cs radiation disrupted HGC-27 cell structures, altered membrane permeability, caused a release of LDH into the cytoplasm, and induced inflammatory and metabolic imbalances. In transmission electron microscopy, we observed that the lipid metabolic disorder and mitochondrial structural damage induced by radiation in the cells were alleviated after pre-treatment or post-treatment with white tea extracts. Polyphenols and flavonoids abundant in white tea extracts activate the antioxidant response element (ARE) via the nuclear factor E2-related factor 2 (NRF2) pathway, significantly enhancing the activities of antioxidant enzymes such as SOD and CAT [24,32]. SOD, as the first line of antioxidant defense, converts superoxide anions (O^2−^) into hydrogen peroxide (H_2_O_2_), which CAT further reduces to water and oxygen, mitigating ROS accumulation [33]. This enzymatic synergy effectively eliminates radiation-induced ROS, suppresses oxidative damage to lipids and proteins, and maintains cellular redox balance. In addition to antioxidation, white tea extracts inhibit radiation-induced apoptosis by suppressing p53-mediated signaling pathways. Radiation typically activates the p53 signaling pathway, upregulating the expression of apoptosis-related proteins such as caspase-3, which disrupts mitochondrial membrane integrity, releasing pro-apoptotic factors and initiating the apoptotic cascade [34]. White tea extracts reduce p53 and caspase-3 expression levels, blocking apoptosis at the molecular level. Active compounds in white tea extracts modulate the Bcl-2/Bax ratio, stabilize mitochondrial membrane potential, and mitigate radiation-induced mitochondrial swelling and crista disruption, preserving cellular energy metabolism and viability [35,36]. Furthermore, these compounds promote DNA repair by regulating the DNA damage response (DDR) pathway, limiting radiation-induced double-strand breaks and enhancing cell survival [37].

Metabolomic analysis revealed that WT-1Y and WT-7Y had distinct metabolite profiles, aligning with their respective radioprotective advantages. WT-1Y demonstrated remarkable efficacy in radiation prevention, attributed to its high levels of rapid free radical scavengers like EGC, procyanidin B4, theanine, and theobromine. EGC is one of the eight types of catechins, and its ability to inhibit radiation-induced cell apoptosis is second to EGCG [38]. It is a potent antioxidant that enhances antioxidant enzyme activity through the NRF2 pathway, providing strong antioxidative protection before radiation exposure [39]. Correlation analysis revealed that EGC was significantly negatively associated with cytotoxicity and apoptotic markers such as LDH, p53, and caspase-3, underscoring its protective role in reducing oxidative stress and inhibiting apoptotic signaling pathways to prevent radiation-induced damage. Procyanidin B4 and other dimeric flavanols abundant in WT-1Y form protective layers prior to radiation exposure, preventing oxidative damage to cell membranes, stabilizing membrane integrity, and reducing direct cellular damage [40,41,42,43]. Furthermore, WT-1Y exhibited a higher phenolic acid content compared to WT-7Y. Among these, methyl gallate is a notable scavenger of hydroxyl radicals (•OH), the primary species generated by water radiolysis, known for its significant cytotoxic effects [7,44]. Oxidative stress often acts as a trigger for inflammation, and inflammatory responses can further amplify ROS production, creating a vicious cycle. Strictinin, a relatively small ellagitannin, mitigates radiation-induced inflammation by inhibiting the NF-κB signaling pathway and reducing the release of pro-inflammatory cytokines, such as TNF-α and IL-6 [45,46]. Additionally, the presence of 1,3,6-Tri-O-galloyll-beta-D-glucose further enhances the anti-inflammatory effect of WT-1Y, including its regulation of inflammatory mediators such as TNF-α, MCP-1, and nitric oxide, preventing the exacerbation of radiation-induced cellular damage [47]. These phenolic acids were also significantly negatively correlated with the levels of p53 and caspase-3 proteins in the pre-treatment groups, indicating their ability to suppress the expression of these apoptosis-related proteins. Supporting this, Huang et al. demonstrated that phenolic acids can inhibit methylglyoxal-induced apoptosis in Neuro-2A cells by stabilizing mitochondrial membrane potential and modulating the Bax/Bcl-2 ratio [48]. Collectively, these properties make WT-1Y particularly suitable for radiation prevention, as its synergistic combination of antioxidative components provided rapid and effective protection for cells prior to radiation exposure.

In contrast, WT-7Y exhibited more enduring protective effects during the post-radiation repair phase compared to WT-1Y. The quercetin content in WT-7Y was significantly higher than that in WT-1Y. Quercetin, a well-known antioxidant in white tea, possesses a unique flavonoid structure characterized by a 2,3 double bond combined with a 4-oxo group in the C ring, facilitating efficient electron delocalization, particularly involving the B ring [49,50]. This extensive resonance significantly enhances its ability to neutralize free radicals. Due to its prolonged storage, WT-7Y exhibited a significant increase in flavanone glycosides, particularly quercetin derivatives such as quercetin-3-glucosylrutinoside and quercetin-3-glucoside. Correlation analysis revealed that these glycosylated flavonoids were positively associated with the activities of CAT in post-treatment with WT-7Y. Glycosylation altered quercetin’s molecular structure, improving water solubility, protecting active sites, enhancing metabolic stability, and extending its half-life in vivo [51]. These modifications enabled a sustained antioxidant release, making WT-7Y particularly effective for prolonged ROS scavenging. However, in the pre-treatment of WT-7Y, glycosylated flavonoids were found to be negatively correlated with pre-CAT, indicating that the protective effect of WT-7Y against radiation is not optimal for this parameter. Additionally, these compounds were also negatively correlated with both pre- and post-SOD levels. As shown in Figure 4, both the radiation and white tea extract treatments resulted in an increase in SOD enzyme activity, which may be related to the cell’s self-defense mechanism. In the damage caused by radiation, SOD enzyme activity is a dynamic indicator, and its increase or decrease alone cannot be used to assess cellular antioxidant capacity. The mechanism by which white tea extracts respond to radiation-induced changes in SOD activity warrants further investigation in the future.

Another key factor in WT-7Y’s post-radiation repair effects is its high caffeine content. As a natural alkaloid, caffeine exhibits both antioxidant and anti-inflammatory properties and inhibits apoptosis via the DDR pathway. Following radiation-induced DNA damage, ATM kinase triggers cell cycle arrest and apoptosis [52]. Caffeine suppresses ATM activity, reducing p53 phosphorylation and γ-H2AX focus formation, thereby limiting DNA double-strand breaks and premature apoptosis [53]. This suppression extended the repair window, enhancing cell survival under radiation exposure. Moreover, WT-7Y contained unique N-ethyl-2-pyrrolidinone-substituted flavan-3-ols (EPSFs), compounds formed during the aging process. EPSFs provide sustained cellular protection and exhibit significant radioprotective potential [54]. Research suggests that EPSFs possess strong antioxidant and anti-inflammatory properties and inhibit the formation of advanced glycation end products (AGEs) [26,55]. The synergistic effects of these bioactive components contributed to the prolonged repair and enhanced protective efficacy of WT-7Y against radiation-induced damage.

White tea extract holds significant promise as a natural radioprotector, addressing critical environmental and public health challenges. Its rich composition, including polyphenols, caffeine, and glycosylated flavonoids, underscore its potential for anti-radiation effects. In this study, WT-1Y and WT-7Y white teas demonstrated distinct radioprotective advantages. The high levels of rapid free radical scavengers present in WT-1Y can quickly react before radiation exposure, eliminating excess free radicals caused by radiation and reducing oxidative damage to the cells. Therefore, under pre-treatment conditions, WT-1Y can effectively protect cells from radiation damage through its rapid free radical scavenging action. On the other hand, WT-7Y has a greater impact on cell viability in the post-treatment phase, mainly due to the presence of its “long-lasting metabolites”. As storage time increases, WT-7Y forms long-lasting metabolites that continue to exert effects during the recovery period following radiation-induced damage. These metabolites help stabilize the cell’s antioxidant system and exert anti-inflammatory effects, extending protection against oxidative stress and significantly enhancing cell viability in the post-treatment phase. Unlike single compounds such as EGCG, which served as the positive control, the diverse metabolites present in white tea extract acted synergistically, enhancing radioprotection through combined antioxidant and anti-apoptotic pathways. This multi-dimensional mechanism highlights the broad potential of white tea extract in a variety of radiation exposure scenarios.

However, it is important to acknowledge some limitations of this study. While we used the cancer cell model, which is widely employed in radiation protection research [56], it may not fully represent the response of normal cells. Nonetheless, cancer cell models can still provide valuable insights into the fundamental mechanisms of radiation-induced damage and the protective effects of white tea extract. Further studies incorporating normal cell lines and in vivo models will be necessary to confirm and extend these findings. Additionally, although our study focused on oxidative stress and apoptosis-related pathways, future research should explore whether white tea extract also influences other mechanisms, such as DNA repair processes, to provide a more comprehensive understanding of its radioprotective potential.

## 4. Materials and Methods

### 4.1. Materials

Epigallocatechin-3-gallate (EGCG, >98%) was purchased from Tauto Biotechnology (Shanghai, China). Fetal bovine serum (FBS) was purchased from Zeta Life (San Francisco, CA, USA). Lactic dehydrogenase (LDH) cytotoxicity assay kit and ROS assay kit were purchased from Beyotime Biotechnology (Shanghai, China). BCA kit, superoxide dismutase (SOD) kit, and catalase (CAT) kit were purchased from Nanjing Jiancheng Bioengineering Institute (Nanjing, Jiangsu, China). Other reagents were purchased from Dalian Meilunbio (Dalian, Liaoning, China).

### 4.2. Preparation of White Tea Sample

Bai Mudan (BMD), a subtype of white tea, stored for 1 year and 7 years, respectively, was obtained from Fuding City (Ningde, Fujian, China). They were extracted in boiling water twice, at 1:30 (*m*/*v*) and 1:20 (*m*/*v*), for 45 min, respectively. The filtrate was mixed and freeze-dried. The white tea extracts (WT extracts) were stored at −40 °C for use; concerning storage time, the 1-year extract was named (WT-1) and the 7-year extract was named (WT-7).

### 4.3. Nontargeted Metabolomics Analysis

Nontargeted metabolomics analysis was performed at the Tea Research Institute, Chinese Academy of Agricultural Sciences, according to a previously reported method [57]. In this study, an ultra-high-performance liquid chromatography–quadrupole/electrostatic field orbitrap mass spectrometer system (UHPLC-Q-Exactive/MS, Thermo Fisher Scientific; Waltham, MA, USA) was used to analyze the metabolome of the tea extracts. The metabolites were separated using an Acquity UPLC HSS T3 column (1.8 μm, 2.1 mm × 100 mm, Waters, Borehamwood, UK). For LC-MS-based metabolic profiling, water with 0.1% (*v*/*v*) formic acid and acetonitrile with 0.1% (*v*/*v*) formic acid were used as mobile phase A and B in both positive (capillary voltage 3.5 kV) and negative (capillary voltage −3.0 kV) electrospray ionization (ESI) mode. The linear profile was as follows: 0 min, 2% B; 0.5 min, 2% B; 8 min, 15% B; 13 min, 35% B; 15 min, 70% B; 16 min, 85% B; 16.5 min, 2% B; and 20 min, 2% B. The column temperature was kept at 40 °C and the flow rate was 0.4 mL/min. The injection volume was 3 μL. The full scan range of the mass-to-charge ratio (*m*/*z*) was set as 100–1200. The capillary temperature was kept at 300 °C. The flow rate of the drying gas was 10 L/min, and the temperature of the drying gas was 350 °C.

The raw data files acquired from the LC-MS analysis were initially imported into the Compound Discoverer 3.2 software (Thermo Fisher Scientific; Waltham, MA, USA) to generate a peak table. The mass width and the retention time width were set at 20 ppm and 0.3 min, respectively. The detailed chemical characterizations of the extracts are shown in Supplementary Material Table S1.

### 4.4. Cell Culture and Treatment

The HGC-27 cells were purchased from the National Collection of Authenticated Cell Cultures. They were grown in a high-glucose DMEM medium with 10% FBS, 100 U/mL penicillin, and 100 μg/mL streptomycin at 37 °C in a humidified atmosphere with 5% CO_2_.

Irradiation was performed at the Institute of Crops and Nuclear Technology, Zhejiang Academy of Agricultural Sciences (Hangzhou, Zhejiang, China), using a ^137^Cs source. The cells with pre- and post-treatments of different concentrations of WT extracts were treated with a total dose of 30 Gy. The pre-treatment involved irradiation after 24 h of incubation with the WT extracts, while the post-treatment involved irradiation before 24 h of treatment with the WT extracts. The dosages of WT-1Y and WT-7Y extracts were 10, 30, and 50 mg/L, respectively. The positive control was 10 mg/L EGCG, the major catechin found in green tea.

### 4.5. Cell Viability Measurement

A CCK-8 kit was applied to measure the cytotoxicity and LDH content in cells. After the pre- and post-treatments with WT extracts, the cell culture medium was collected to detect LDH content, and the adherent cells were used to detect cell viability. The collected medium was mixed with an LDH kit at 37 °C for 30 min. The absorption was read using an ELISA microplate reader (Thermo, Multiscan MK3, Waltham, MA, USA) at 490 nm and 680 nm. A total of 100 μL of 10% CCK-8 solution was added to each well and was incubated at 37 °C for 1 h. The absorption was detected at 450 nm.

### 4.6. ROS Level Detection

The intracellular ROS levels in HGC-27 cells were measured using 2′,7′-dichlorodihydrofluorescein diacetate (DCFH-DA). The cells were incubated in 10 μM DCFH-DA at 37 °C for 20 min and washed with PBS three times to remove excess DCFH-DA. The fluorescence of different treatments was detected using a multi-functional microplate reader (Agilent Technologies, BioTek Synergy H1, Hayward, CA, USA) at 488 nm/525 nm (Ex/Em).

### 4.7. Antioxidant Enzyme Detection

After 15 min of cell lysis, the lysates were centrifuged at 14,000 rpm at 4 °C for 5 min. The sediment was discarded, and the supernatant was transferred to new EP tubes [58]. The detection of SOD and CAT activity was carried out according to the instructions of the reagent kits (Jiancheng, Nanjing, China).

### 4.8. Ultrastructure Analysis of Cell

After pre- and post-treatments of WT extracts, cells were collected by centrifugation at 3000 rpm for 5 min. Supernatant was discarded, and cells were fixed with 2.5% glutaraldehyde in PBS (0.1 M, pH = 7.0) overnight. According to the previously reported methods, ultra-thin stained sections were made and observed in Hitachi Model H-7650 TEM [59].

### 4.9. Immunological Fluorescence Assay

After pre- and post-treatments of WT extracts, cells were washed three times with PBS, then fixed with 4% paraformaldehyde for 15 min. Immunological fluorescence assay was performed at Hubei Biossci Co., Ltd. (Wuhan, Hubei, China), and 0.1% TritonX-100 (15 min, room temperature) was used for membrane rupture. After blocking in 10% donkey serum (20 min, room temperature), corresponding primary (overnight, 4 °C) and secondary (30 min, 37 °C) antibodies were added sequentially. Cell nucleus was stained with DAPI in darkness (10 min, room temperature). Scanner (3DHistech, Pannoramic SCAN II, Budapest, Hungary) was used for image capture.

ImageJ-win 64 software (version: 1.53k) was applied to analyze fluorescence intensity.

### 4.10. Data Analysis

Statistical analysis of experimental data was conducted using SPSS 26.0 software. A Student’s *t*-test was used to analyze significant differences. Data were represented as mean ± standard deviation (SD). PCA, PLS-DA plots, and heatmaps were drawn by OmicShare (https://www.omicshare.com/tools/) (accessed on 1 May 2024).

## 5. Conclusions

This study highlights the significant radioprotective potential of white tea, demonstrating its ability to mitigate ^137^Cs radiation-induced cellular damage through multiple mechanisms. Enriched with diverse bioactive compounds, white tea effectively reduced oxidative stress, enhanced antioxidant defenses, and protected mitochondrial function via anti-apoptotic pathways, providing a comprehensive defense against radiation exposure. Metabolomic analysis identified differential metabolites caused by storage time, and most of them were strongly associated with radiation protection. WT-1Y, characterized by high levels of rapid free radical scavengers, such as EGC, procyanidin B4, and phenolic acids, offered robust antioxidant protection prior to radiation exposure. Conversely, WT-7Y, enriched with long-lasting metabolites, such as quercetin-3-glucosylrutinoside and DNA repair facilitators like caffeine, excelled in post-radiation repair.

In summary, white tea extracts from different storage periods offered a multi-layered defense mechanism, underscoring their potential for broad applications in diverse radiation exposure scenarios. White tea extracts may offer a sustainable and natural solution for radioprotection and functional food development, with significant promise for safeguarding individuals exposed to radiation. These findings pave the way for future research and practical applications on white tea, contributing to the broader goal of mitigating radiation-related health risks.

## Data Availability

The data that supports the results is confidential but can be made available upon reasonable request from other researchers.

## Supplementary Materials

The following supporting information can be downloaded at https://www.mdpi.com/article/10.3390/molecules30071448/s1.
